# FN3 Domain Displaying Double Epitopes: A Cost-Effective Strategy for Producing Substitute Antigens

**DOI:** 10.3389/fmolb.2021.742617

**Published:** 2021-11-04

**Authors:** Yao Ruan, Shuangying Chao, Xuejun Hu, Longzhen Lu, Yue Lin, Qian Wang, Yang Zheng, Junming Li, Ning Ding

**Affiliations:** ^1^ Xi’an International Medical Center Hospital, Xi’an, China; ^2^ Medical College, Dalian University, Dalian, China; ^3^ Xi’an Engineering Technology Research Center for Cardiovascular Active Peptides, Xi’an, China; ^4^ Department of Clinical Laboratory, Yuhuangding Hospital, Yantai, China

**Keywords:** FN3, substitute antigen, epitope, NT-ProBNP, *Escherichia coli*

## Abstract

Construction of substitute antigens based on alternative scaffold proteins is a promising strategy in bioassay technology. In this study, we proposed a strategy for constructing substitute antigens derived from 10th human fibronectin type III (FN3) using two peptide epitopes of terminal pro-brain natriuretic peptide (NT-proBNP) as an example. The base sequences encoding the two antigenic epitopes of NT-proBNP were recombined into the FG loop region and the C-terminus of FN3, fused by 4 GS or polyN linker. The fusion proteins (named FN3-epitopes-4GS and FN3-epitopes-polyN, respectively) were expressed and purified cost-effectively using an *Escherichia coli* expression system. The immunoreactivity of recombinant substitutes was preliminarily confirmed by western blot analysis using epitope-specific antibodies. The sandwich enzyme-linked immunosorbent assay demonstrated that either FN3-epitopes-polyN or FN3-epitopes-4GS was highly sensitive, and FN3-epitopes-polyN exhibited better kinetics to specific antibodies than FN3-epitopes-4GS, showing a linear dose-response relationship in the concentration range of 0.06–12.85 ng/ml, which suggest that the polyN linker was more suitable for constructing the FN3-based substitute antigens compared to the 4 GS linker. Furthermore, the serum stability test and differential scanning calorimetry analysis showed that the recombinant FN3-epitopes-polyN maintained the original stability of FN3. Therefore, it was confirmed that FN3 could be engineered to construct a stable biomacromolecular substitute for displaying double epitopes of antigen proteins, such as NT-proBNP. In summary, a cost-effective strategy to produce NT-proBNP substitute antigens with good immunoreactivity and physicochemical stability was established in this work, which may provide potential uses for the production of other substitute antigens in the future.

## Introduction

The 10th human fibronectin type III (FN3) domain has become one of the most widely used non-antibody scaffolds for engineering novel binding proteins (Koide et al*.*, 1998; Gill and Damle, 2006; Chandler and Buckle, 2020). Several FN3-based biological molecules have been developed for therapeutic and diagnostic applications (Shingarova et al., 2018; Chandler and Buckle, 2020; Sirois et al., 2020). The structural simplicity and superior biophysical characteristics of FN3 offer advantages for the construction of stable and novel biomacromolecules. FN3 contains no disulfide bonds or free sulfhydryl groups, contributing to its stability at high temperatures (Tm = 87°C) and reduced environment (Plaxco et al., 1996; Bloom and Calabro, 2009). Furthermore, the structure of the three flexible surface-exposed loops (BC, DE, and FG) of FN3 are similar to that of antibody variable districts, and the change in amino acid sequences in the BC and FG loops did not disturb the stability of macromolecular derivatives of FN3 (Koide et al*.*, 1998; Liu et al*.*, 2019). FN3 lacks cysteine residues, suggesting high-level expression in the cytoplasm of bacteria, such as *Escherichia coli*. In previous studies, we efficiently expressed monobodies and receptor proteins based on FN3 as a protein scaffold in *E. coli* (Ding et al., 2019; Zhu et al., 2020).

The binding of an antibody to its target is mostly highly specific. Therefore, one antibody can only recognize a specific part of the antigen called an “antigenic epitope” (Sela-Culang et al., 2013). The display of antigenic epitopes is a critical step in the production of substitute antigens *in vitro*. Studies have shown that linear epitopes are determined by the amino acid sequences rather than the 3D shapes, unlike the discontinuous conformation epitopes (Kringelum et al., 2013). Therefore, instead of constructing full-length antigens to function as immunogens, a feasible alternative is to conjugate the specific linear antigenic epitopes to some macromolecular carriers. For example, terminal pro-brain natriuretic peptide (NT-proBNP) has been widely used for the reliable diagnosis of heart failure and cardiac dysfunction, but it is easily degraded and cannot be used as a standard antigen for *in vitro* diagnostic tests (Semenov et al., 2017; Cao et al., 2019; Mueller et al., 2019). As the exact linear epitopes responsible for the immunity of NT-proBNP are well studied (Hughes et al., 1999; Seferian et al., 2008), it is possible to manufacture short linear-epitope peptides of NT-proBNP using stable scaffold proteins, such as FN3, to facilitate the production, purification, and detection at a low cost via the *E. coli* expression system.

Using NT-proBNP as an example, we developed a strategy for producing stable recombinant antigen proteins by employing the FN3 scaffold protein to display two epitopes via the *E. coli* expressing system. The selected epitope sequences 12–21 and 62–73 of NT-proBNP were reconstructed in the FG loop and C-terminus of FN3, respectively, to explore the immunoreactivity of recombined macromolecules by quantitative sandwich ELISA detection. A polyN (SSNNNNNNNNNN) linker or 4 GS (GGGGS) linker was introduced to ensure a better connection between the two epitopes; thus, two different fusion proteins, FN3-epitopes-4GS and FN3-epitopes-polyN, were synthesized. The results demonstrated that the FN3-based biomacromolecular substitutes with the same immunoreactivity of NT-proBNP could be rapidly and efficiently produced in the *E. coli* expression system, avoiding the complexity and high cost of expression in eukaryotic cell lines or synthesis by chemical methods. In summary, this study demonstrated that FN3 could be used as a scaffold protein to display the two specific antigenic epitopes to produce the corresponding stable substitute antigens of NT-proBNP cost-effectively via the *E. coli* system, with good biophysical characteristics of high stability and immunoreactivity*.*

## Materials and Methods

### Bacterial Strains and Chemicals

*E. coli* BL21 (DE3) used for protein production experiments was obtained from Sangon Biotech (Shanghai, China). Kanamycin, IPTG, TMB, and HEPES were obtained from Sigma-Aldrich (United States). The monoclonal antibody, HRP-conjugated mouse anti-human NT-proBNP 4NT1C-13G12, and mouse anti-human NT-proBNP 4NT1-15C4 were purchased from Hy-Test (Finland). HisTrap™HP (1 ml) for His-tagged protein purification was obtained from GE Healthcare (United States). PVDF membrane and 6x-His tag monoclonal antibody were purchased from Thermo Fisher Scientific (United States). All other chemicals and solvents were purchased from Sangon Biotech (Shanghai, China).

### Plasmid Construction

The pET28a (+) vector and restriction enzymes were obtained from TaKaRa Biotechnology (Dalian, China). Plasmids were constructed using standard molecular techniques. The genes encoding scaffold protein FN3, whose FG loop and C-terminus were replaced with two epitopes of NT-proBNP, were chemically synthesized by Nanjing GenScrip Tech Ltd. (Nanjing, China), and cloned into the pET28a (+) vector by *Nco*I and *Hind*III sites. Moreover, the genes encoding 4 GS linker or polyN linker were introduced upstream of epitope 62–73 generating two similar but different plasmids [named pET28a (+)-FN3-epitopes-4GS and pET28a (+)-FN3-epitopes-polyN]. The successful insertion of linker and epitope DNA into the plasmids was confirmed by DNA sequencing.

### Homology Modeling

To obtain the 3D structures of terminal linear-epitopes, homology modeling was performed using RaptorX (http://raptorx.uchicago.edu/) for the protein secondary structure prediction and template-based tertiary structure modeling. The amino acid sequence of FN3 was retrieved from the RCSB PDB database (PDB:1FNA) and used as a target for homology modeling. The alignment of two protein structures was performed in PyMOL (The PyMOL Molecular Graphics System; Version 1.5.0.3).

### Expression and Purification of Proteins

To produce recombinant FN3 proteins (generally named as FN3-epitopes) that display the two epitopes of NT-proBNP, the plasmid pET28a (+)-FN3-epitopes-4GS or pET28a (+)-FN3-epitopes-polyN was transformed into *E. coli* BL21 (DE3) cells. A single colony was picked and grown overnight at 37°C in 3 ml Luria-Bertani (LB) medium containing 50 μg/ml kanamycin (Kan). This culture was inoculated at a ratio of 1/100 into 50 ml LB media and further grown at 37°C with shaking until the OD600 reached 0.4–0.6, followed by addition of 0.2 mM isopropyl β-D-1-thiogalactopyranoside (IPTG) to induce the expression of FN3-epitopes at 25°C with shaking. Expression was allowed to continue for 6 h. After harvesting the cells by centrifugation, the crude product was extracted by ultrasonication and centrifugation at 12,000 g, and the proteins of interest were purified using HisTrap™HP (1 ml). In addition, to optimize protein purification, the supernatant of the cell lysate containing target proteins was first heated at 40°C for 10 min. Thereafter, the precipitate was removed at high speed to obtain the supernatant for importing to the HisTrap™HP. The expression and purification of target proteins were monitored by sodium dodecyl sulphate–polyacrylamide gel electrophoresis (SDS-PAGE) or western blot analysis. Protein concentrations were determined by bicinchoninic acid (BCA) assay kit (Thermo Fisher Scientific, United States).

### Thermal Denaturation Experiment

After ultrasonication and centrifugation, the supernatants from bacterial extracts containing the proteins of interest were used for the thermal denaturation experiments. In brief, 80 μL of the supernatant was heat-denatured separately at different temperatures (37, 40, 45, 50, 55, 60, 65, and 70°C) for 10 min. The supernatant and pellet were separated by centrifugation at 12,000 g. Finally, the results were analyzed using SDS-PAGE gels.

### Western Blot Analysis

The pellets corresponding to 1 OD unit of cells were resuspended in 0.1 ml PBS with loading buffer and boiled for 10 min. After cooling to room temperature, 0.01 ml samples were separated by 15% SDS-PAGE gels and transferred onto a polyvinylidene fluoride (PVDF) membrane. The PVDF membrane-bound to proteins was first incubated with primary antibody HRP-conjugated mouse anti-human NT-proBNP 4NT1C-13G12 (specifically binds to human NT-proBNP_13-20_) or mouse anti-human NT-proBNP 4NT1-15C4 (specifically binds to human NT-proBNP_63-71_). HRP-conjugated goat anti-mouse IgG was used as the secondary antibody. All blots were visualized using a Chemidoc™ XRS + system with Image Lab™ image capture software (Bio-Rad, United States).

### Detection of Soluble Proteins

The pellets corresponding to 10 OD units of cells were harvested and resuspended in 1 ml PBS. The resuspended cells were treated by ultrasonication for 8 min of 3 s pulse with 2 s intervals at 200 W, and centrifugation at 12,000 g, and the precipitate was resuspended in 1 ml PBS. Thereafter, 0.1 ml of the resuspended precipitate or the supernatant was mixed with loading buffer and boiled for 10 min. Finally, the soluble expression of target proteins was detected by SDS-PAGE, and western blot analysis using 6x-His tag monoclonal antibody.

### Evaluation of Antigen-Binding Capacity

The antigen-binding capacity of the FN3-epitopes was assessed using a double-antibody sandwich ELISA. The 96-well plates were coated with mouse anti-human NT-proBNP 4NT1-15C4 (3.5 μg/ml) as capture antibodies and incubated for 3 h at 37°C. Bovine serum albumin (BSA; 5%) was added to each well and incubated at 4°C overnight. Serially diluted purified protein was added to each well and incubated for 3 h at 37°C. HRP-conjugated mouse anti-human NT-proBNP 4NT1C-13G12 (0.35 μg/ml) served as a detection antibody and was incubated for 1.5 h at 37°C. The colored reaction product was developed by addition of 3,3′,5,5′-Tetramethylbenzidine (TMB) and the reaction was terminated after 30 min by 1 M sulphuric acid. The absorbance of each well was measured at a UV wavelength of 450 nm. Data were analyzed using Prism GraphPad software. Curves were adjusted using a sigmoid dose-response (variable slope) equation, Y=Bottom + (X^Hillslope)*(Top-Bottom)/(X^HillSlope + EC50^HillSlope)), where X is the logarithm of concentration and Y is the response. Y starts at Bottom and goes to Top with a sigmoid shape.

### Differential Scanning Calorimetry

DSC detection was performed using a standard procedure (Cui et al., 2017). The purified proteins were diluted to a final concentration of 200 μM in PBS. The samples were dialyzed in 10 mM HEPES, 50 mM NaCl, and 2 mM Tris-HCl, and the concentrations were adjusted to 100 mM (pH 7.25). Samples were heated from 40°C to 120°C at a scan rate of 90°C/h and analyzed via VP-DSC (MicroCal LLC, United States). All samples were assayed by cooling and reheating and comparing the recovery of cal values derived from the integration of the transition peak.

### Serum Stability Test of FN3-Epitopes-polyN

To evaluate the *in vitro* stability of the recombinant proteins in human plasma, the purified FN3 and FN3-epitopes-polyN protein were spiked into the pooled plasma to a final concentration of 50 μg/ml. The plasma sample was incubated at 37°C for 80 h. Equal volumes of samples were collected at different time points and then detected by western blotting using 6x-His tag monoclonal antibody.

## Results

### Structures of FN3-Epitopes

**FIGURE 1:**
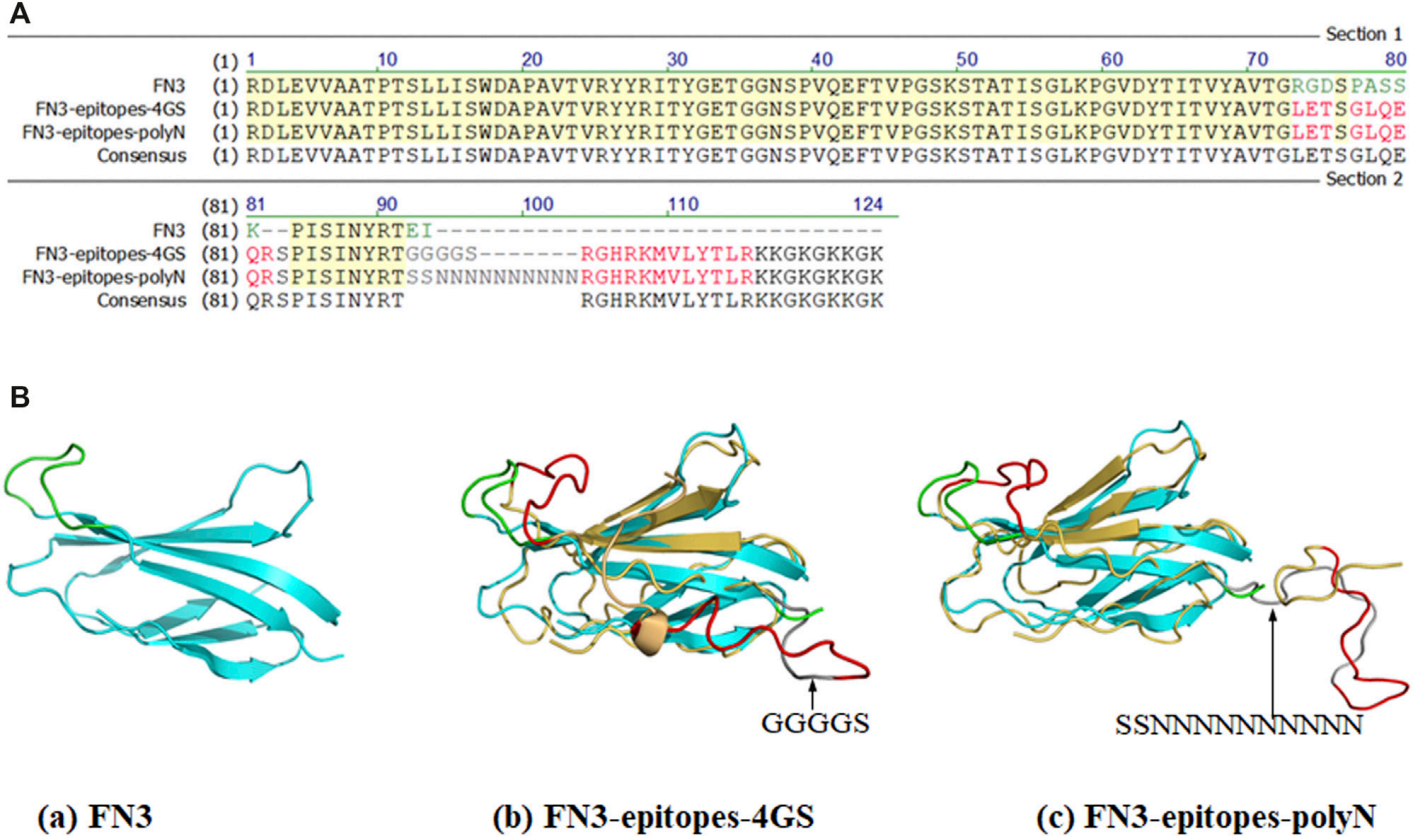
Partial sequences and structures of FN3, FN3-epitopes. (**A**) The partial sequences of FN3 protein engineered with the NT-proBNP epitopes. The green letters represent FG loop (RGDSPASSK) areas or C-terminus (EI) of FN3, and the red letters represent the inserted NT-proBNP epitope 12–21 (LETSGLQEQR) in the FG loop or epitope 62–73 (RGHRKMVLYTLR) in C-terminus. The grey letters represent the inserted 4 GS linker or polyN linker. (**B**) The 3D structures of FN3 (cyan) and modeling structures of recombinant FN3 with NT-proBNP epitope sequences (epitope 12–21 and epitope 62–73 were shown with red colored lines) at different sites, and the 4 GS linker or polyN linker was shown with dark grey coloring lines. The backbones of the FN3 are colored in cyan, which are superposed with the homology models in yellow.

To produce a biomacromolecule for displaying double antigenic epitopes of NT-proBNP, we chose the two positions (FG loop and C-terminus) of FN3 that are relatively far from each other in spatial structure as implantation sites to avoid possible interaction between the two epitopes. The amino acid sequence RGDSPASSK of the FG loop region of FN3 was replaced by epitope 12–21 of NT-proBNP. Furthermore, the epitope 62–73 of NT-proBNP was recombined with the C-terminus of FN3, and the linker KKGKGKKGK was added to facilitate coupling with other molecules, such as Biotin and peroxidase. Specifically, we introduced polyN or 4 GS linkers to fuse the above two epitopes in the FN3 domain to ensure the normal functions of the target proteins, resulting in the construct named FN3-epitopes-4GS or FN3-epitopes-polyN (Figure 1).

### Expression of FN3-Epitopes

**FIGURE 2:**
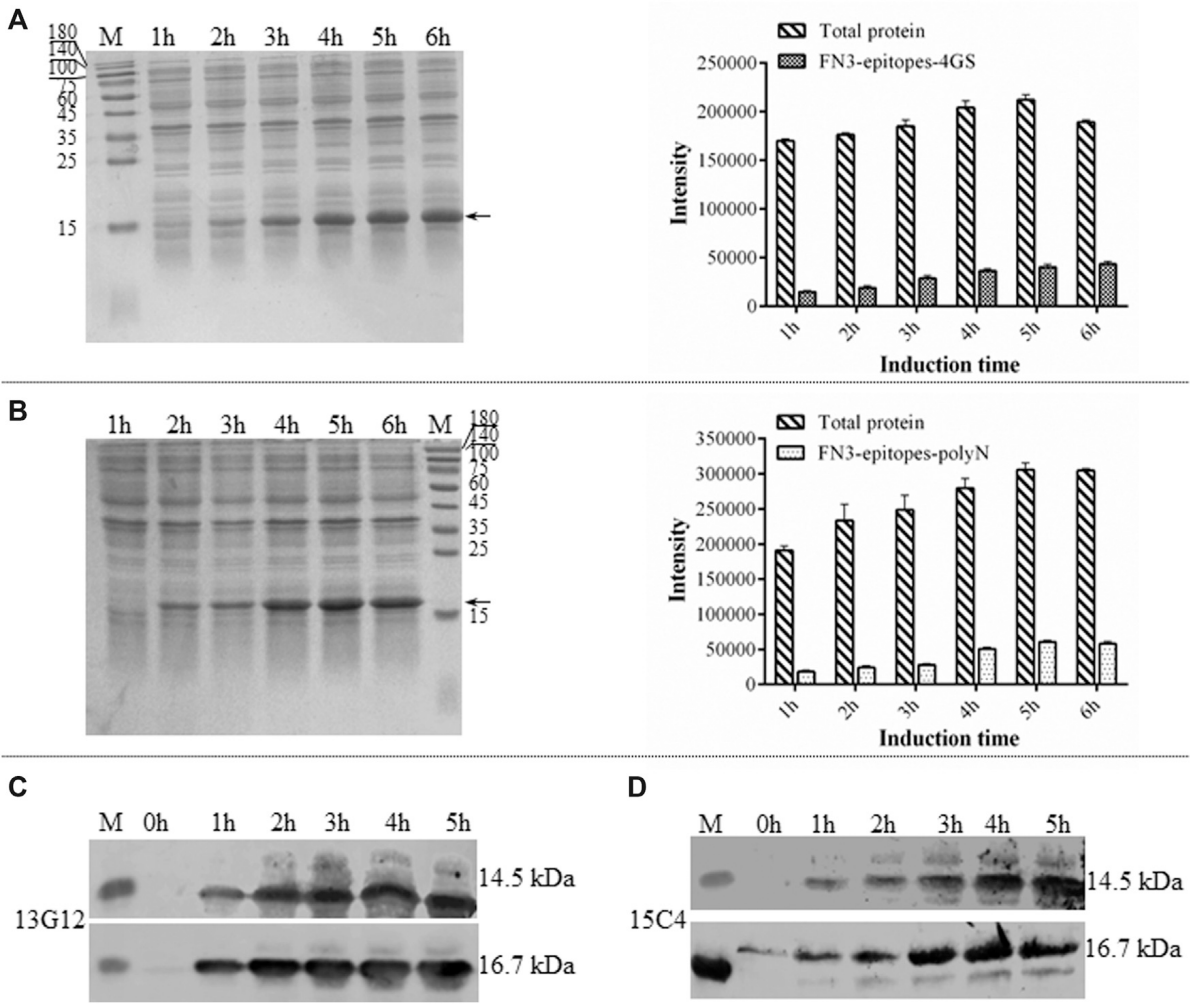
The expression of recombinant FN3-epitopes in *E. coli*. (**A, B**) SDS-PAGE analysis of the expression of protein FN3-epitopes-4GS (A) and FN3-epitopes-polyN (B) after induction for 1–6 h. Arrow indicates the target protein FN3-epitopes-4GS (14.5 kDa) or FN3-epitopes-polyN (16.7 kDa), and each sample of the total bacterial extract loaded on the gels is from 1 OD unit of cells. The signal intensity calculated from SDS-PAGE images in part A and B, respectively. Data correspond to averages of three independent experiments. Error bars represent standard deviations. (**C, D**) Western blot analysis of the expression of FN3-epitopes-4GS (upper panel) and FN3-epitopes-polyN (lower panel) at the indicated induction time points using HRP-conjugated mouse anti-human NT-proBNP 4NT1C-13G12 (C) or mouse anti-human NT-proBNP 4NT1-15C4(D), specific to the amino acid residues 13–27 and 61–76 of NT-proBNP, respectively. M: molecular marker.

Due to its simple folding, high thermostability, and lack of disulfide bonds, the derivatives of FN3 could be expressed at a high level in the cytoplasm of *E. coli* BL21 (DE3). The recombinant FN3-epitopes could be recognized by commercial monoclonal antibodies specific to different regions of NT-proBNP, corresponding to amino acid residues 13–27 and 61–76. The recombinant proteins FN3-epitopes-polyN (16.7 kDa) and FN3-epitopes-4GS (14.5 kDa) were expressed under the control of the IPTG-inducible T7 promoter (Figure 2A, Figure 2B), and both reached the highest level with similar amounts (approximately 20% of total protein) after 5 hours of induction. Western blot analysis showed that the recombinant proteins FN3-epitopes-polyN and FN3-epitopes-4GS could bind to the selected antibodies specifically (Figure 2C, Figure 2D).

### Purification of FN3-Epitopes

**FIGURE 3:**
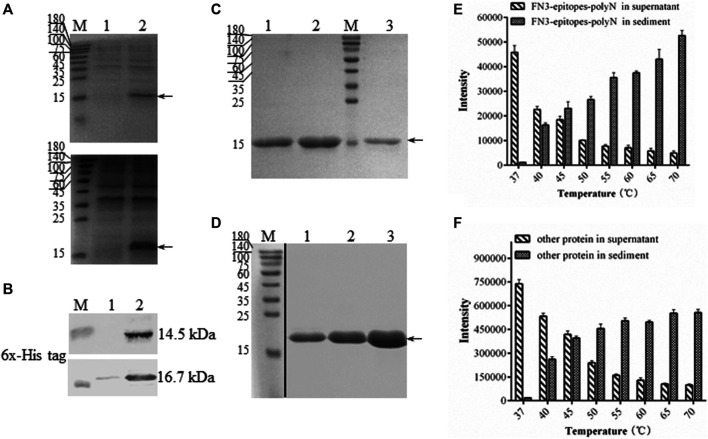
The solubility and purification of recombinant FN3-epitopes. **(A)** SDS-PAGE analysis of the soluble expression of FN3-epitopes-4GS (upper panel) and FN3-epitopes-polyN (lower panel) in *E. coli* BL21 (DE3). Arrow indicates the target protein FN3-epitopes-4GS (14.5 kDa) or FN3-epitopes-polyN (16.7 kDa). Lane 1: the precipitate of total protein lysates; Lane 2: the supernatant of total protein lysates. (**B**) Western blot analysis of the soluble expression of FN3-epitopes-4GS (upper panel) and FN3-epitopes-polyN (lower panel) in *E. coli* BL21 (DE3). Lane 1: the precipitate of total protein lysates; Lane 2: the supernatant of total protein lysates. (**C, D**) SDS-PAGE analysis of the isolation of FN3-epitopes-4GS (C) and FN3-epitopes-polyN (D) via the HisTrap™ purification. Arrow indicates the target protein FN3-epitopes-4GS (14.5 kDa) or FN3-epitopes-polyN (16.7 kDa). Lanes 1–3 in (C): protein from three tubes eluted sequentially using 60 mM imidazole; lanes 1–3 in (D): protein from three tubes eluted sequentially using 80 mM imidazole. M: molecular marker. (**E, F**) Histogram depicting the thermal degradation of FN3-epitopes-polyN (E) and other proteins except FN3-epitopes-polyN (F). Signal intensities calculated from SDS-PAGE images in Supplementary Fig. 2. Data correspond to averages of three independent experiments. Error bars represent standard deviations. M: molecular marker.

To ensure the yields of target proteins, we explored the intracellular expression characteristics of FN3-epitopes in *E. coli*. The results showed that FN3-epitopes-polyN and FN3-epitopes-4GS were expressed mainly in a soluble form in *E. coli* BL21 (DE3) (Figure 3A, Figure 3B). However, the purification efficiency of protein FN3-epitopes-4GS (∼9.5 mg/L of bacterial culture) was significantly lower than that of protein FN3-epitopes-polyN (∼21 mg/L of bacterial culture) (Figure 3C, Figure 3D; for detailed information, see Supplementary Figure S1) under the similar level of protein expression, as shown in Figure 2A and Figure 2B. These results indicate that the polyN linker is more efficient than the 4 GS linker in the purification of fusion proteins displaying double antigenic epitopes based on FN3.

Furthermore, we performed a thermal denaturation experiment to detect the level of degradation of FN3-epitopes-polyN protein under different temperature conditions (37–70°C) to facilitate the purification process by affinity chromatography. The results revealed that the FN3-epitopes-polyN protein transferred gradually from the supernatant to precipitate as the temperature increased. Approximately 30% of the heteroprotein precipitated at 40°C, whereas 60% of the target protein still existed in the supernatant at this temperature (Figure 3E, Figure 3F; for detailed information, see Supplementary Figure S2). Taking advantage of this thermal stability, we optimized the purification process by heating the supernatant of FN3-epitopes-polyN cell lysate to 40°C before importing it to the metal-chelate chromatography, which resulted in more high-quality purified proteins (∼40 mg/L) (for detailed information, see Supplementary Figure S3). The purity of the recombinant protein FN3-epitopes-polyN was approximately 100% according to SDS-PAGE analysis (Figure 3D, Supplementary Figure S3); thus, these purified proteins could be used as calibrators or standards without further purification steps.

### High Antigenic Reactivity of FN3-Epitopes

**FIGURE 4:**
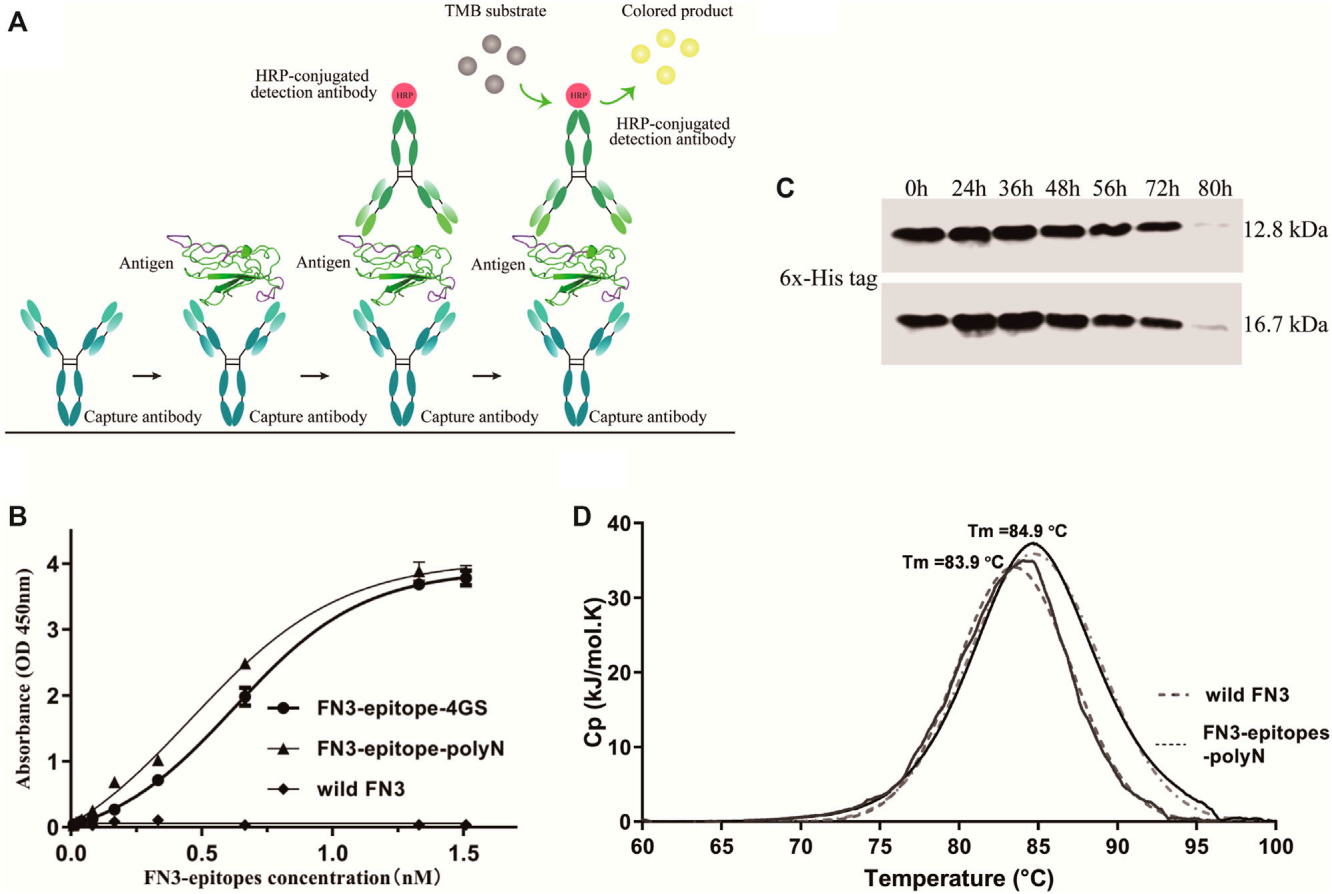
The immunoreactivity and stability detection of the recombinant FN3-epitopes-polyN. (**A**) The scheme of sandwich ELISA. (**B**) The immunoreactivity detected by sandwich ELISA. Capture antibody: mouse anti-human NT-proBNP 4NT1-15C4 (3.5 μg/ml), Detection antibody: HRP-conjugated mouse anti-human NT-proBNP 4NT1C-13G12 (0.35 μg/ml), Substrate solution: TMB. (**C**) The serum stability of protein FN3 (upper panel) and FN3-epitopes-polyN (lower panel) tested by western blotting. (**D**) The thermostability detected by DSC, revealing similarly high Tm values for FN3-epitopes-polyN (Tm = 83.9°C) and the wild FN3 (Tm = 84.9°C).

Using wild FN3 as a negative control, double-antibody sandwich ELISA was used to systematically assess the immunoreactivity of recombinant FN3-epitopes-polyN and FN3-epitopes-4GS. Figure 4A represented the scheme of sandwich ELISA, and the results showed that both recombinant FN3-epitopes had good linear relationships with the specific antibodies, in the range of 0.06–12.85 ng/ml for FN3-epitopes-polyN, and the *r* ^2^ values of standard curves were greater than 0.99 (Figure 4B, Supplementary Figure S4). Furthermore, FN3-epitopes-polyN showed better affinity at low concentrations compared to that of FN3-epitopes-4GS. In summary, the double epitopes of NT-proBNP could be successfully displayed based on the FN3 domain and maintained their original high antigenic reactivity. Thus, considering the above advantages of easy purification and better affinity, this study focused on analyzing recombinant protein FN3-epitopes-polyN.

### Stability of FN3-Epitopes-polyN *in vitro*

To clarify whether the fusion of the two additional epitopes has any impact on the stability of recombinant proteins, we further verified the serum stability and thermal stability of FN3-epitopes-polyN. Serum stability detection of recombinant FN3-epitopes-polyN was performed in normal human plasma *in vitro*. The results showed that the FN3-epitopes-polyN degraded after 80 h of incubation with normal human plasma at 37°C, similar to the un-engineered protein FN3 (Figure 4C). The results of DSC revealed that only a single peak was found in the detection of protein FN3-epitopes-polyN (Tm = 83.9°C), with a similar Tm value of wild FN3 (Tm = 84.9°C), indicating that the purified protein FN3-epitopes-polyN maintained the stability of un-engineered FN3 (Figure 4D). The above stability tests indicated that the recombinant protein FN3-epitopes-polyN would be a highly stable substitute for the antigen NT-proBNP.

## Discussion

This study demonstrated an effective strategy to construct a substitute antigen based on the skeleton protein FN3, which was proven to successfully display two different epitopes of the appointed antigen NT-proBNP. To the best of our knowledge, no studies have reported engineered FN3 as a synthetic substitute antigen, and this study verified that the introduction of the two epitopes did not affect the stability of FN3 and maintained the original immunoreactivity of the epitopes.

This study indicated that FN3 could be used to develop stable substitute antigens in detection kits. The necessary features, such as compatibility with many molecules and special structural features, have made FN3 a particularly established scaffold for developing non-antibody binding domains (Hantschel, 2017; Sha et al., 2017; Chandler and Buckle, 2020). The N- or C-terminus of FN3 is considered a regular and reasonable region to be engineered as antigenic epitopes because it is flexible and could be extended without perturbing the secondary conformations of nearby structures. Moreover, as reported, the amino acid sequences of the FG loop can tolerate highly artificial introduced-sequence diversities without changing the stability or solubility of derivatives (Koide et al*.*, 1998; Ramamurthy et al*.*, 2012; Liu et al*.*, 2019), which is consistent with our stability assay and thermal denaturation experiments of recombinant substitute antigens (as shown in Figure 3, Figure 4, and Supplementary Figure S2). In particular, as shown in Figure 1, we selected the C-terminus and FG loop, which are far away from each other in the crystal structure of FN3 to reduce the impacts of local conformation between the two neighboring antigenic epitopes. Furthermore, compared to the 4 GS linker, the polyN linker exhibited a better ability to depart the two antigenic epitopes in the secondary conformation, which is necessary to reconstruct the substitutes of antigen calibrator for the sandwich ELISA detection methods (Chen et al., 2013) (as shown in Figure 1 and Figure 4B).

As the sandwich ELISA for quantitative detection of NT-proBNP has been considered a classical standard technique, and the standard antigens must contain at least two specified antigenic epitopes, FN3 was engineered with the two epitopes of NT-proBNP, linear epitopes 12–21 and 62–73, separated by a flexible linker in this study (Figure 1). The two epitopes were selected because it has been reported that antibodies specific to the central part of the NT-proBNP molecule (peptides 28–45 and 46–60 of sequence) being utilized in immunoassays scarcely conjugated with the antigens in human blood samples (Schellenberger et al., 2006). This is explained by O-glycosylation of the central part of NT-proBNP molecules, both in human blood and recombinantly expressed in eukaryotic cells (Schellenberger et al., 2006; Kuwahara et al., 2018). Therefore, it is recommended to use an antibody specific to the N- or C-terminal parts of NT-proBNP for precise quantitative measurements of serum NT-proBNP, which, according to our results, is the same as the process of engineering FN3 as a substitute antigen and needs further investigation.

Based on the results, this study proposes a cost-effective strategy to produce a sensitive and stable recombinant substitute antigen of NT-proBNP at a low cost using an *E. coli* expression system. It is a promising method for using FN3-epitopes-polyN to detect NT-proBNP because of its high sensitivity, low cost, specificity, rapid response, and low labor requirements. In our assay, the paring capture and detection antibodies, 4NT1-15C4 and 4NT1C-13G12, exhibited high affinity to the two epitopes engineered in C-terminal and FG loop respectively. The FN3-epitopes-polyN molecule can recognize endogenous and recombinant human NT-proBNP at the standard range of 0–770 pmol/L (Supplementary Figure S4, 1 pmol/L = 16.7 pg/ml, refers to FN3-epitopes-polyN that is detected by the sandwich ELISA), as sensitive (the limit of detection: 3.5 pmol/L) as other literature (Seferian et al., 2008; Coumans et al., 2017) and the widely used clinical detecting kits, e.g. NT-proBNP ELISA KIT (Cat.No.SK-1204, BIOMEDICA). Comparison of commercial elecsys proBNP assay (Roche Diagnostics) especially, the standard FN3-epitopes-polyN is easy to obtain (for expression, purification, and quantitative detection in one and a half working days), stable and convenient to perform, leading to a simple and rapid ELISA detection of serum NT-proBNP levels in 3 h.

For the sake of the good biophysical characteristic of high stability and immunoreactivity of the recombinant FN3-epitopes-polyN, our methodology has special advantages of low cost and high efficiency. For the efficient detection of clinical NT-proBNP, not only the sandwich ELISA formats’ requirement of using a stable standard antigen and a pair of antibodies with high affinities, but also the cost and time invested for running the assay have been explored in this study. Despite not reaching a superior detectability, the assay based on FN3-epitopes-polyN as the standard antigen is sufficient to satisfy the clinical needs, and it shows advantages derived from the low cost-benefit ratio, simplicity, and safety by using the commercial TMB substrate. Although there are competitive assays employing radioactive labels with better detectability, however, most of which raises the drawbacks related to the handling of reagents and waste management.

Another important consideration is the low cost-benefit ratio to expand the clinical applications of using FN3-epitopes-polyN for NT-proBNP detection. As the results presented here, through the optimized chromatography purification process, the level of contaminating proteins was significantly reduced by up to 40% after the whole-cell lysates were heated. It is not easy to purify native NT-proBNP as a standard antigen as the concentrations of NT-proBNP or BNP in human serum are rather low and often contaminated with high concentrations of other proteins. It is expensive to produce antigen protein NT-proBNP from eukaryotic cell lines owing to high-cost cell culture and the easy degradation of NT-proBNP (Dasgupta et al., 2003; Yeo et al., 2003). In contrast, our study has provided a cost-effective means of harvesting stable recombinant FN3-epitopes-polyN proteins directly from the heated lysates of *E. coli* BL21 (DE3) cells, reaching a yield of at least 40 mg/L in a 2 L shake-flask cultivation.

In conclusion, using the two selected epitopes of NT-proBNP as an example, our proposed approach provides a simple and efficient strategy to produce biomacromolecular substitutes by engineering the FN3 scaffold in *E. coli*. This work makes it possible to produce a large-scale stable detection calibrator in *E. coli* cost-effectively. This method may have general implications on producing other artificial recombined proteins, particularly for synthesizing unstable complex proteins in humans.

## Data Availability

The raw data supporting the conclusions of this article will be made available by the authors, without undue reservation.
