# Supplementary material for: FN3 Domain Displaying Double Epitopes: A Cost-Effective Strategy for Producing Substitute Antigens
Source: Front Mol Biosci. 2021 Nov 4;8:742617. doi: 10.3389/fmolb.2021.742617 (PMC8607273; doi:10.3389/fmolb.2021.742617)
Supplement: Supplementary file 1 [file DataSheet1.pdf]

I. Amino acid sequence of FN3-epitopes-4GS and FN3-epitopes-polyN

1. Amino acid sequence of FN3-epitopes-4GS

MA**HHHHHH**GGGAQVSDVPRDLEVVAATPTSLLISWDAPAVTVRYYRITYGE
6 His

TGGNSPVQEFTVPGSKSTATISGLKPGVDYTITVYAVTG**LETSGLQEQR**SPISI
NT-proBNP epitope 12-21

NYRT**GGGGSRGHRKMVLYTLR**KKGKGKKGK*
4GS NT-proBNP epitope 62-73

2. Amino acid sequence of FN3-epitopes-polyN

MA**HHHHHH**GGGAQVSDVPRDLEVVAATPTSLLISWDAPAVTVRYYRITYGE
6 His

TGGNSPVQEFTVPGSKSTATISGLKPGVDYTITVYAVTG**LETSGLQEQR**SPISI
NT-proBNP epitope 12-21

NYRT**SSNNNNNNNNNNRGHRKMVLYTLR**KKGKGKKGK*
polyN NT-proBNP epitope 62-73

II. Supplementary Figures

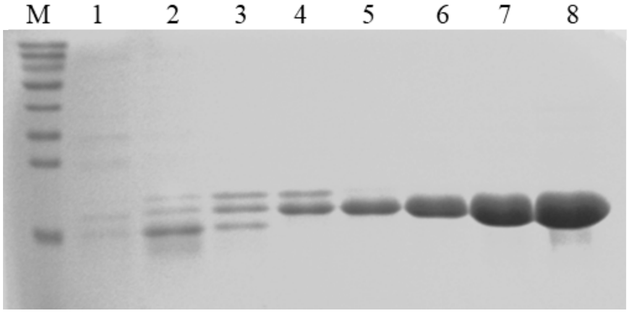

Supplementary Fig. 1 The whole picture of Fig. 3D. lane 1–4: proteins from four tubes eluted sequentially using 60 mM imidazole, lane 5–8: proteins from four tubes eluted sequentially using 80 mM imidazole, M: molecular marker.

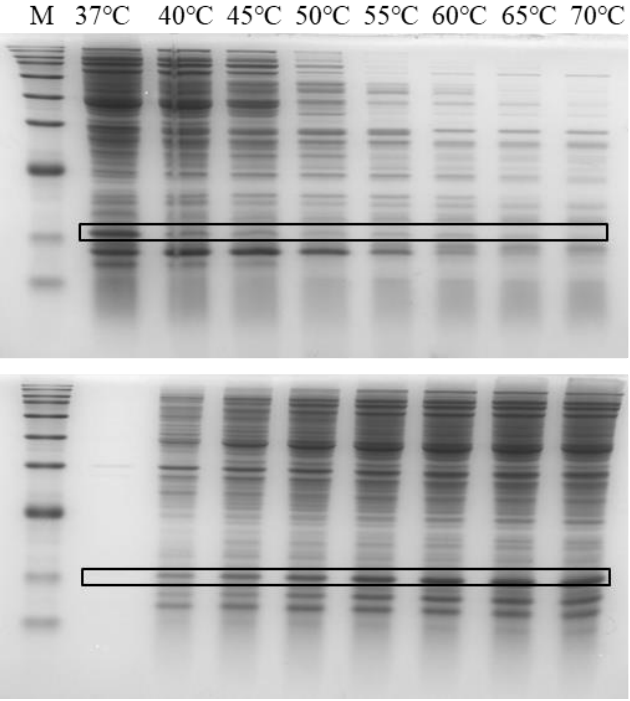

Supplementary Fig. 2 SDS-PAGE analysis of the thermal degradation detection under different temperature conditions. The target protein is shown in the box. Upper panel: total protein in supernatant. Lower panel: total protein in precipitate. M: molecular marker.

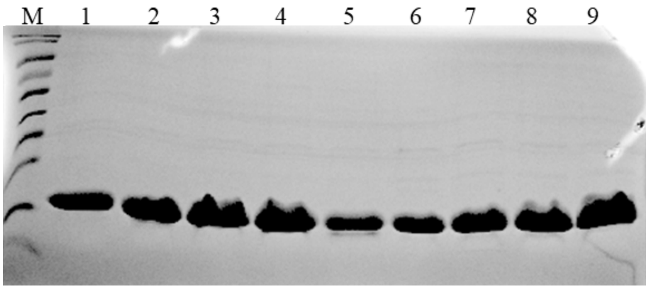

Supplementary Fig. 3 SDS-PAGE analysis of the purified FN3-epitopes-polyN by process optimization. lane 1–5: proteins from five tubes eluted sequentially using 60 mM imidazole. lane 6–9: proteins from four tubes eluted sequentially using 80 mM imidazole. M: molecular marker.

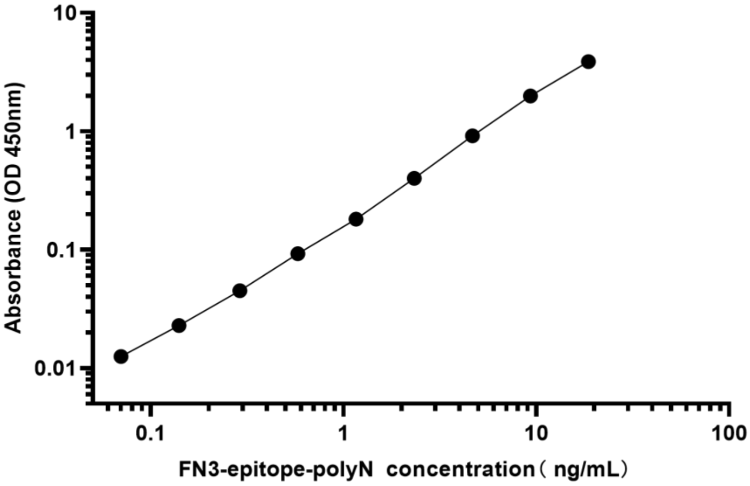

Supplementary Fig. 4 The antigenic reactivity detected by sandwich ELISA. Capture antibody: mouse anti-human NT-proBNP 4NT1-15C4 (3.5 μg/mL), Detection antibody: HRP-conjugated mouse anti-human NT-proBNP 4NT1C-13G12 (0.35 μg/mL), Substrate solution: TMB, the limit of detection: 0.06 ng/mL.
